# Sphincter preservation in patients with low rectal cancer: striking the right oncological balance

**DOI:** 10.1007/s12672-021-00400-1

**Published:** 2021-03-15

**Authors:** Federico Luvisetto, Awad Shamali, Marieke L. W. Rutgers, Karen Flashman, Jim S. Khan

**Affiliations:** 1 Department of Colorectal Surgery, Portsmouth Hospitals NHS Trust, Southwick Hill, Portsmouth, PO6 3LY UK; 2 Honorary Associate Professor, School of Health, Education, Medicine and Social Care, Anglia Ruskin University, Cambridge, UK

**Keywords:** Rectal cancer, Surgery, Low anterior resection, Abdominoperineal excision, Cancer recurrence, Survival

## Abstract

**Background:**

The surgical treatment options for low rectal cancer patients include the Abdominoperineal Resection and the sphincter saving Low Anterior Resection. There is growing evidence towards better outcomes for patients being treated with a Low Anterior Resection compared to an Abdominoperineal Resection.

**Objective:**

The aim of this study was to evaluate the short term and oncological outcomes in low rectal cancer treatment.

**Design:**

This is a retrospective cohort study of prospectively collected data.

**Setting:**

Rectal cancer patients from a single center in the United Kingdom.

**Patients:**

Patients included all low rectal cancer patients (≤ 6 cm from the anal verge) undergoing Low Anterior Resection or Abdominoperineal Resection between 2006 and 2016.

**Outcome measures:**

To identify differences in postoperative complications and disease free and overall survival.

**Results:**

A total of 262 patients were included for analysis (Low Anterior Resection n = 170, Abdominoperineal Resection n = 92). Abdominoperineal Resection patients were significantly older (69 versus 66 years), had lower tumours (3 versus 5 cm), received more neo-adjuvant radiation, had longer hospital stay and more complications (wound infections and wound dehiscence). Low Anterior Resections had a significantly higher number of harvested lymph nodes (17 versus 12) however there was no difference in nodal involvement and R0 resection rate. No significant difference was found for recurrence, overall survival and disease free survival.

**Limitation:**

Retrospective review of cancer database and single center data.

**Conclusion:**

In the treatment of low rectal cancer Abdominoperineal Resection is associated with higher rates of postoperative complications and longer hospital stay compared to the Low Anterior Resection, with similar oncological outcomes.

## Introduction

Once the classical paradigm for rectal cancer treatment was; ‘The lower the cancer, the worse its prognosis’. Nowadays equivalent oncological outcomes can be achieved for all rectal cancer patients, no matter its height and even with sphincter preserving options. The first one to describe the APER or Abdomino Perineal Excision of Rectum was Sir W. E. Miles in 1908 and for a century this procedure remained the gold standard for the treatment of cancers of the lower rectum. Over the last few decades the surgical management of distal rectal cancer has shifted from the traditional APER or Miles procedure to low or even ultralow sphincter-preserving anterior resections (LAR, uLAR) [1, 2]. These changes have been facilitated by the widespread application of the Total Mesorectal Excision (TME) principle [3], better stapling devices [4], recognition of the prognostic importance of an involved circumferential resection margin (CRM) (rather than the distal resection margin) and the increasing use of neoadjuvant (chemo)radiotherapy for locally advanced rectal tumours [5, 6].

At present, a safe margin is considered to be a distal mural margin of 1–2 cm [7]. For low rectal tumors a one cm distal margin is accepted because distal intramural spread occurs over 1 cm in only 4% to 10% of the cases [8, 9]. This knowledge, that a distal margin of 1 cm is considered to be oncologically safe, means that in theory an anastomosis can be made at almost any level in the pelvis [6]. Whenever safe margins cannot be achieved, a non-restorative procedure is still the treatment of choice. Such is the case when the CRM is involved, with involved Extra Mural Vascular Invasion (EMVI), locally advanced lesions with poor response to neoadjuvant therapy and with tumours with external sphincter or levator ani muscle invasion [10, 20].

Consequently, sphincter-preserving surgery has now become a priority with many units publishing APER to LAR ratio of 1:3 or 1:4 in recent times [11–13]. Furthermore the intersphincteric resection technique offers reconstructive surgery in patients with a tumor close to or even in the anal canal without compromising local control and survival.

Radical oncological surgery still remains the main goal of the surgical treatment; however functional outcome, both short and long term, must be considered in the balance. Several studies have shown that quality of life in patients treated with APER is not inferior to LAR, despite the presence of a permanent colostomy [14]. In case of a low or ultra-low anastomosis the possibility of incontinence or low anterior rectum syndrome (LARS) should also be considered [15]. Thus it still remains an argument of debate whether those patients with lower rectal cancer eligible for surgical treatment or better treated with the non-restorative APER or sphincter saving LAR.

The aim of this analysis is to compare outcomes between sphincter preserving surgery and APER in a single center series of low and ultralow rectal cancer patients, stratified by stage, looking at oncological adequacy of resection, morbidity and short-term results.

## Patients and methods

Between September 2006 and December 2016, all patients undergoing TME surgery with curative intent for low rectal cancer (up to 6 cm from the anal verge) at Queen Alexandra Hospital in Portsmouth (UK) were included in this retrospective analysis of a prospectively collected database.

All included patients underwent digital rectal examination, sigmoidoscopy and staging with computer tomography (CT) of the chest and abdomen and magnetic resonance imaging (MRI) of the pelvis and/or endoanal ultrasound for preoperative staging. Patients were offered surgery following a consensus decision by the local Colorectal Multidisciplinary Team (cr-MDT) after deciding the need for neoadjuvant chemoradiotherapy (CRT). Long course chemoradiotherapy was given to T4 rectal cancers or those with a threatened or involved circumferential resection margin (CRM) on MRI. Curative resection was performed either by an open approach or laparoscopically according to the TME principle. Curative being defined as no macroscopic cancer left within the abdomen at time of surgery.

The choice between an open and laparoscopic procedure was dependent upon the surgeon’s experience and preference for that particular patient. APER or LAR decision was made after detailed consultation with the patient and analysis of their preoperative scans and examination findings with regards to the tumor height and distance from the anal verge.

### Data collection and study definitions

The obtained database collected information on demographics, score of the American Society of Anesthesiology (ASA), pre-operative clinical stage, neo-adjuvant chemotherapy or radiotherapy, patho-histological data, peri-operative mortality (defined as either in-hospital mortality or death within 30 days of surgery in case of earlier discharge), length of hospital stay and post-operative complications using the Clavien-Dindo classification. Survival data was last updated on the 30th of June 2017. Disease-free survival was defined as the time from the date of primary treatment (surgery) to the date of first recurrence, be it local, systemic or both if they had occurred 6 months apart. Overall survival was defined as the time from the date of the primary treatment to the date of death. Patients were divided into 2 groups according to the type of procedure, the LAR and the APER group.

### Study outcomes

The primary study outcome was the long-term oncological outcomes (survival, recurrence and disease free survival) in patients who had low rectal cancer surgery. Secondary outcome was short term outcomes, including peri-operative complications, following rectal surgery.

### Statistical analysis

Statistical analyses was performed using SPSS version 22.0 (SPSS Inc., Chicago, IL, USA). Data was expressed as median with inter-quartile ranges. Intergroup comparisons were made using a Mann–Whitney U test for continuous variables or chi squared or Fishers exact test for categorical variables. A difference with a p value < 0.05 was considered significant. Kaplan–Meier survival plots and the log rank test were used to compare disease-free and overall survival between the two groups. Cox regression analysis was performed on all available factors.

## Results

### Clinicopathologic features

**Fig. 1:**
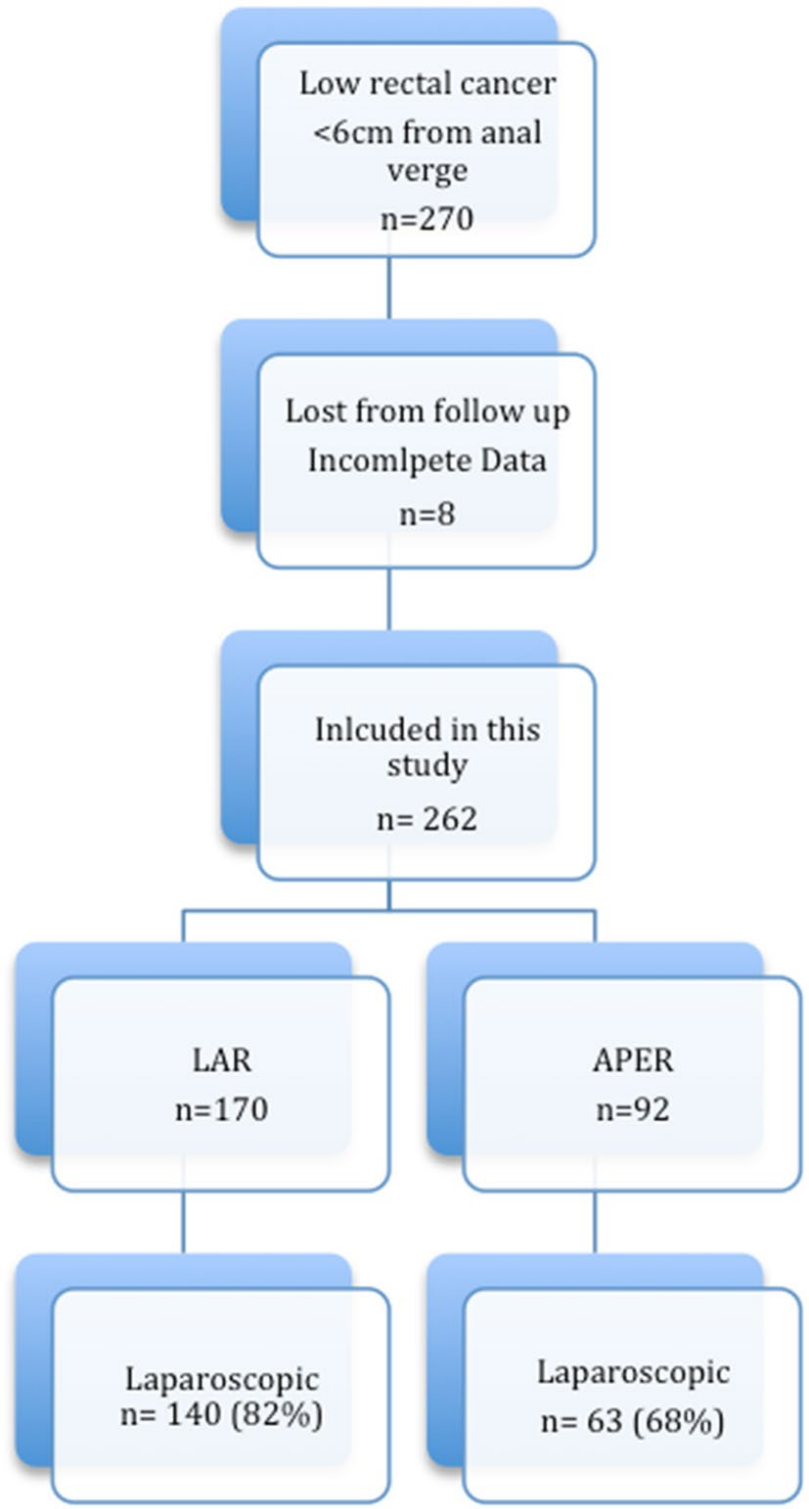
Flow chart of case distribution

A total of 270 patients were identified to have surgical treatment for low rectal cancer during the study period. Eight patients were excluded from further analysis due to incomplete data and lack of follow up. Thus, data from 262 patients who underwent a curative surgical TME resection for low rectal cancer were reviewed (Fig. 1). Of these, 170 patients underwent LAR and 92 patients were treated with APER. The majority of the resections were performed laparoscopically (LAR 82.4%, APER 68.5%), by highly experienced surgeons assisted by a senior registrar/fellow. Patient characteristics and treatment details are summarized in Table 1. Overall, the majority of patients was male (72%) with no significant difference in the male/female distribution between the groups. Patients in the APER group were significantly older, p = 0.040, with no significant difference in ASA score and BMI.

**Table 1:** Patient demographics

Variables	LAR	APER	p value
Number (%)	170 (64.9)	92 (35.1)	
Age, years median (interquartile range)	66 (59–75)	69 (62–77)	*0.040*
Gender M/F	110/60	53/39	0.287
BMI, median (interquartile range)	26 (23–29)	27 (25–31)	0.146
ASA score			
ASA 1	19 (11.2)	4 (4.5)	0.089
ASA 2	124 (72.9)	62 (69.7)	0.392
ASA 3	27 (15.9)	23 (25.8)	0.101
DAV, median (interquartile range)	5 (4–6)	3 (2–4)	*< 0.001*
Clinical stage			
I	9 (5.3)	1 (1.1)	0.173
II	79 (46.5)	36 (39.1)	0.297
III	62 (36.5)	38 (41.3)	0.506
IV	7 (4.1)	7 (7.6)	0.257
Pre-operative radiation	41 (24.1)	47 (51.0)	*0.001*
Neoadjuvant chemotherapy	30 (17.6)	28 (30.4)	*0.020*
Laparoscopic approach	140 (82.4)	63 (68.5)	*0.013*

Values in italics are statistically significant

Continuous variables expressed as medians and interquartile ranges. Nominal variables expressed as absolute numbers with percentages in parenthesis

*APR* abdominoperienal resection, *LAR* low anterior resection, *BMI* body mass index, *ASA* American Society of Anesthesiologists classification, *DAV* distance from anal verge

The APER group had a significantly lower tumor height (3 cm in APER vs. 5 cm in LAR, p < 0.001). When comparing the distribution of the pre-operative MRI T and N stage, the two groups showed no significant difference. However, significant more APER patients had pre-operative radio- and chemotherapy, (p = 0.001 and 0.020 respectively).

### Oncological and long-term outcomes

**Fig. 2:**
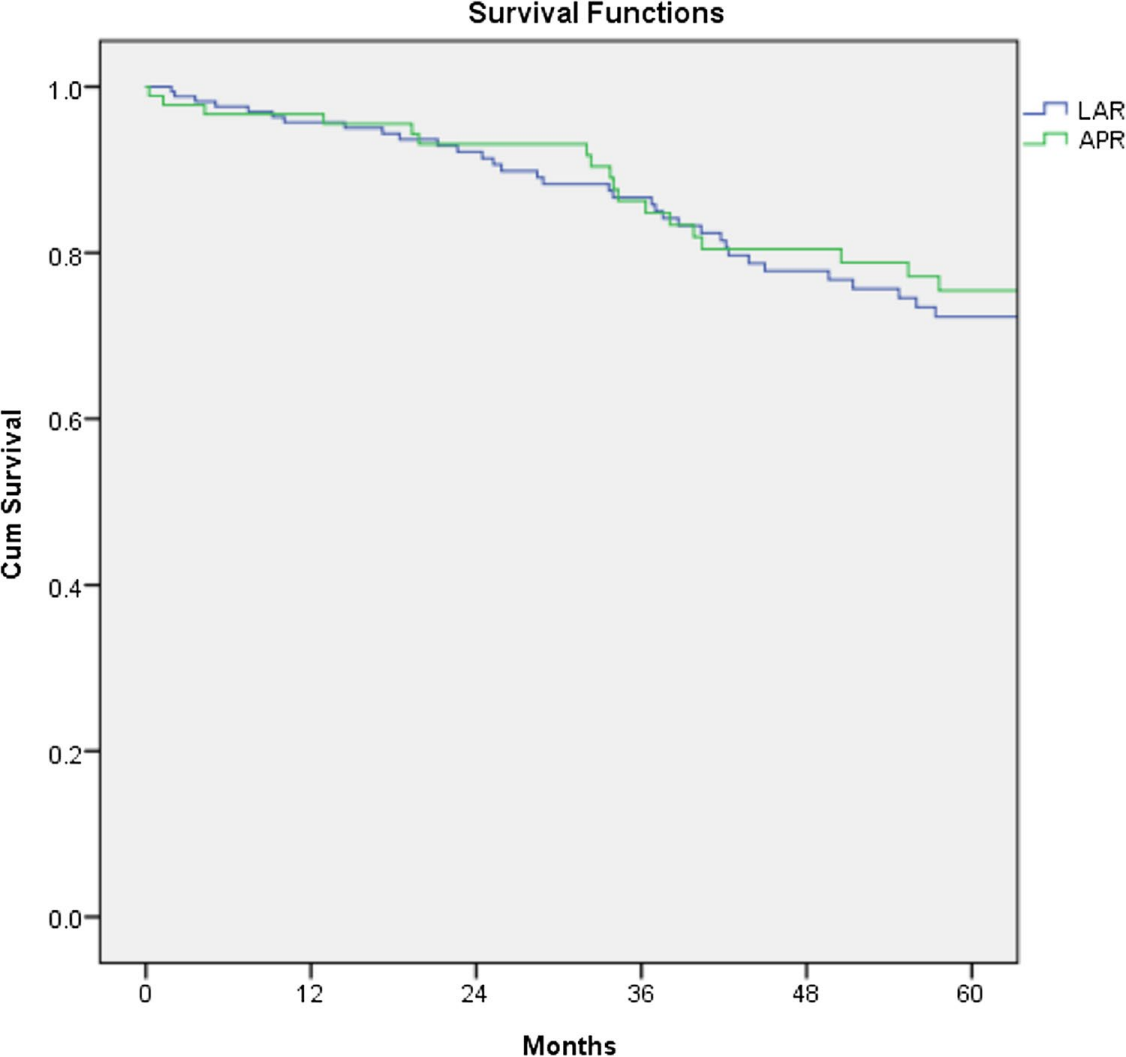
Kaplan–Meier plot revealing the survival of LAR compared to APR following resection for adenocarcinoma. No significant difference between the two groups was demonstrated (log Rank, p = 0.514).

Median tumor size was similar in both groups, p = 0.253. The absolute R1 rate (microscopic tumor infiltration of the margin) was higher in the APER group, albeit not reaching statistical significance, p = 0.085. The LAR group had a significantly higher number of harvested lymph nodes (LN) compared to the APER group, p < 0.001. However, there was no difference in lymph node positivity between the two groups, see Table 2. The median survival was 55 months in the APER group versus 49 months for the LAR group, although non-significant, p = 0.514 (Fig. 2).

**Table 2:** Pathological characteristics and long-term outcomes

Variables	LAR	APER	p value
Median tumour size, mm (interquartile range)	36 (25–50)	35 (27–40)	0.253
Number of Lymph nodes, median (interquartile range)	17 (12–22)	12 (8–18)	*< 0.001*
Pathological lymph nodes stage			
N0	118 (69.8)	60 (66.7)	0.492
N1	33 (19.5)	18 (20.0)	1.000
N2	18 (10.7)	12 (13.3)	0.549
Microscopic tumour infiltration of the margin (R1)	9 (5.3)	11 (12.0)	0.085
Pathological stage			
I	20 (11.8)	5 (5.4)	0.123
II	65 (38.5)	37 (40.2)	0.791
III	77 (45.6)	43 (46.7)	0.897
IV	2 (1.2)	5 (5.4)	0.053
Post-operative chemotherapy	46 (27.1)	20 (21.7)	0.374
Post-operative radiation	2 (1.2)	2 (2.2)	0.614
Local recurrence	7 (4.1)	4 (4.3)	1.000
Distance recurrence	23 (13.5)	18 (19.6)	0.215

Value in italic is statistically significant

**Fig. 3:**
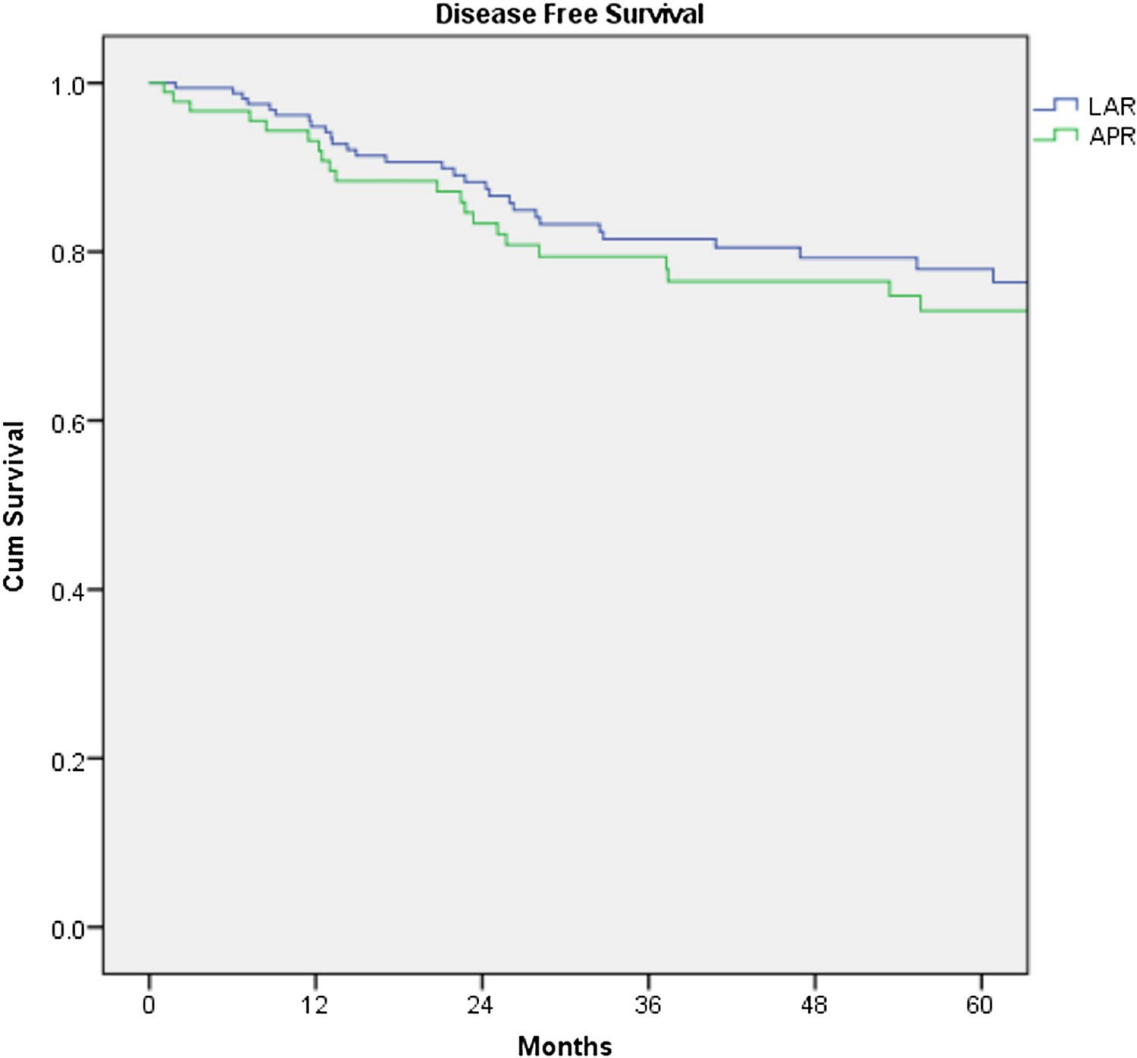
Disease free survival probability depending on type of surgical procedure, log Rank = 0.455

**Fig. 4:**
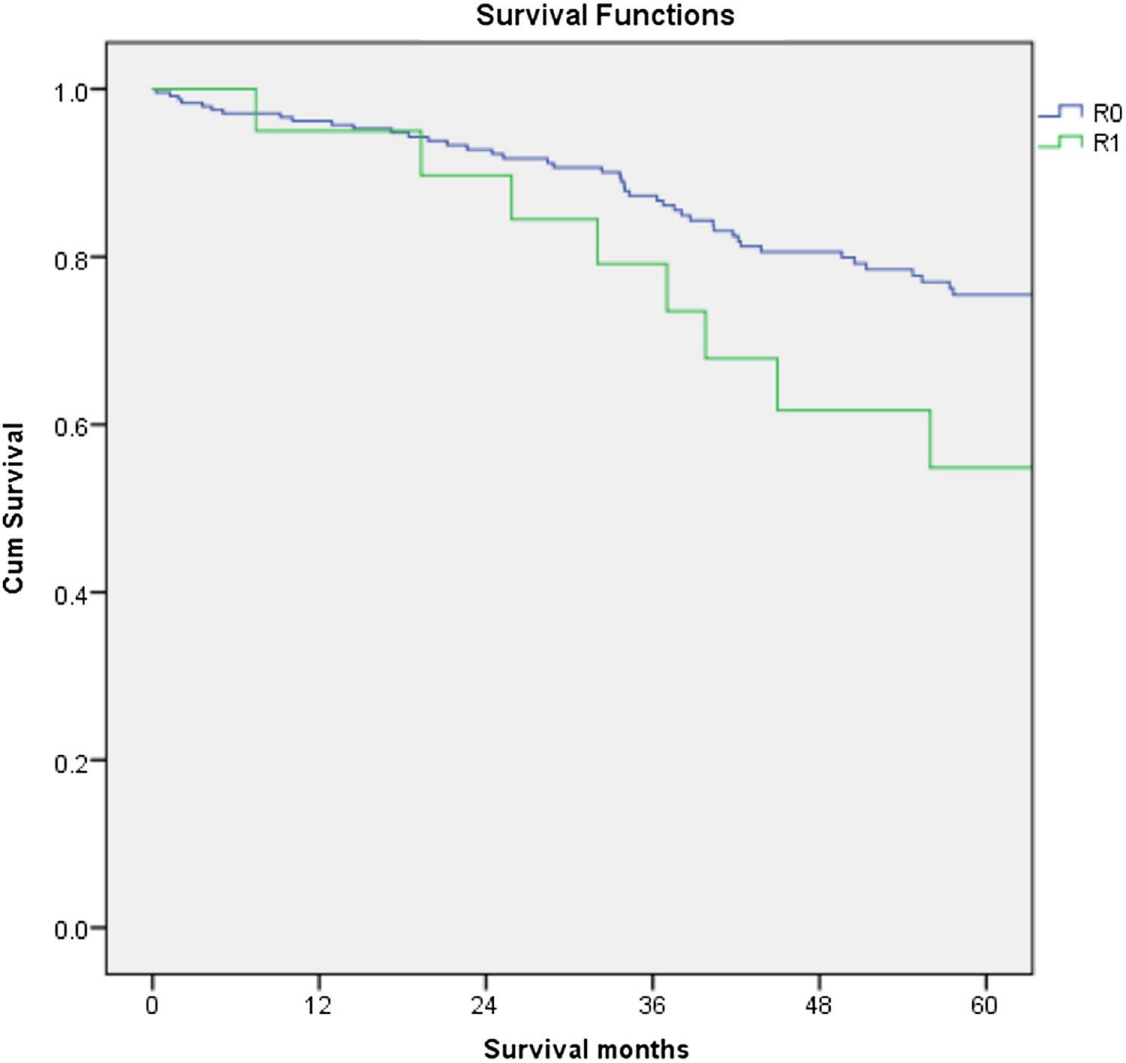
Cumulative survival for patients undergoing a curative resection for low rectal cancer comparing patients with R0 resection to patients with R1 resection, log Rank = 0.023

**Fig. 5:**
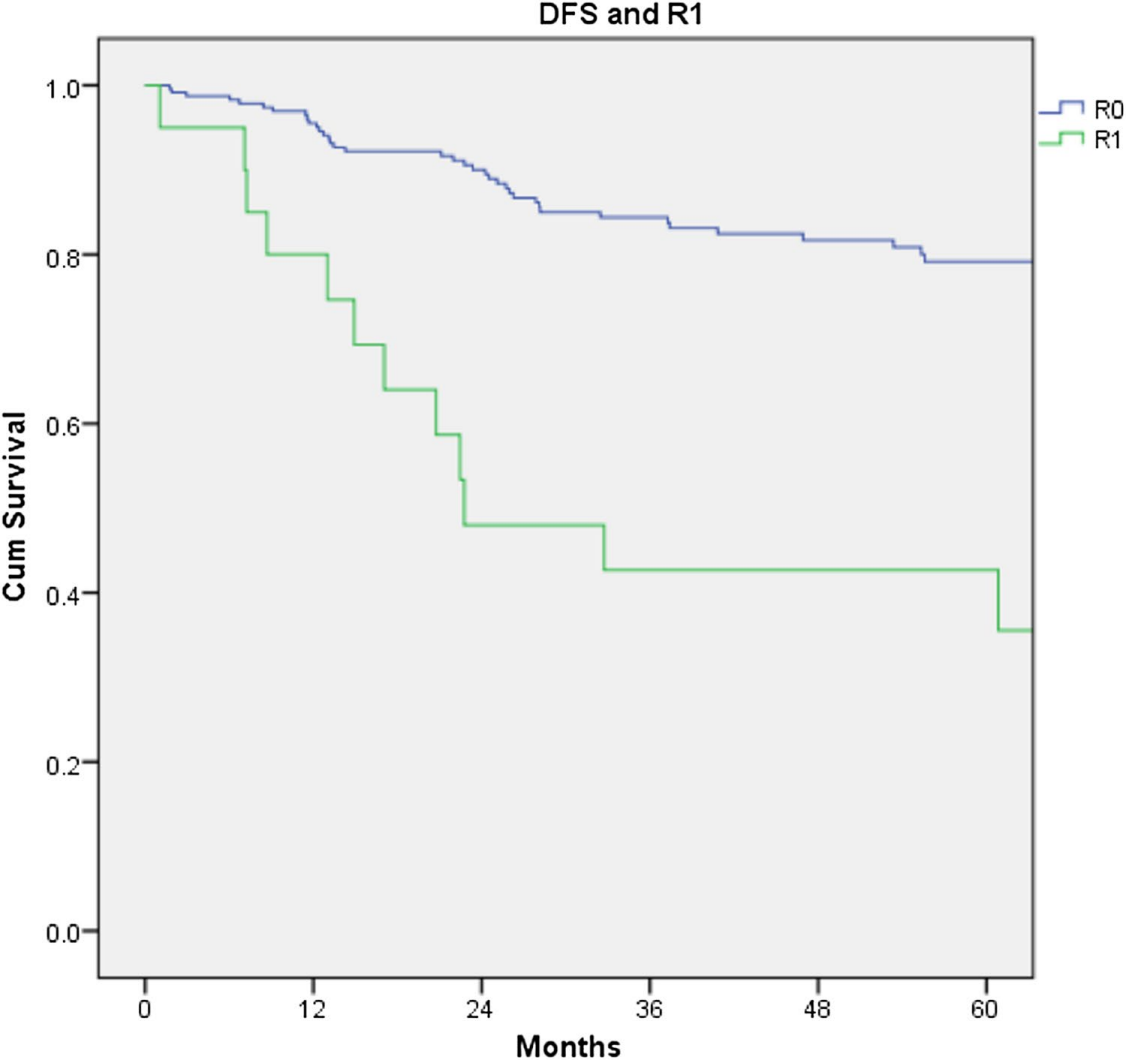
Disease free survival probability depending on R1 margin resection, log Rank < 0.001

**Fig. 6:**
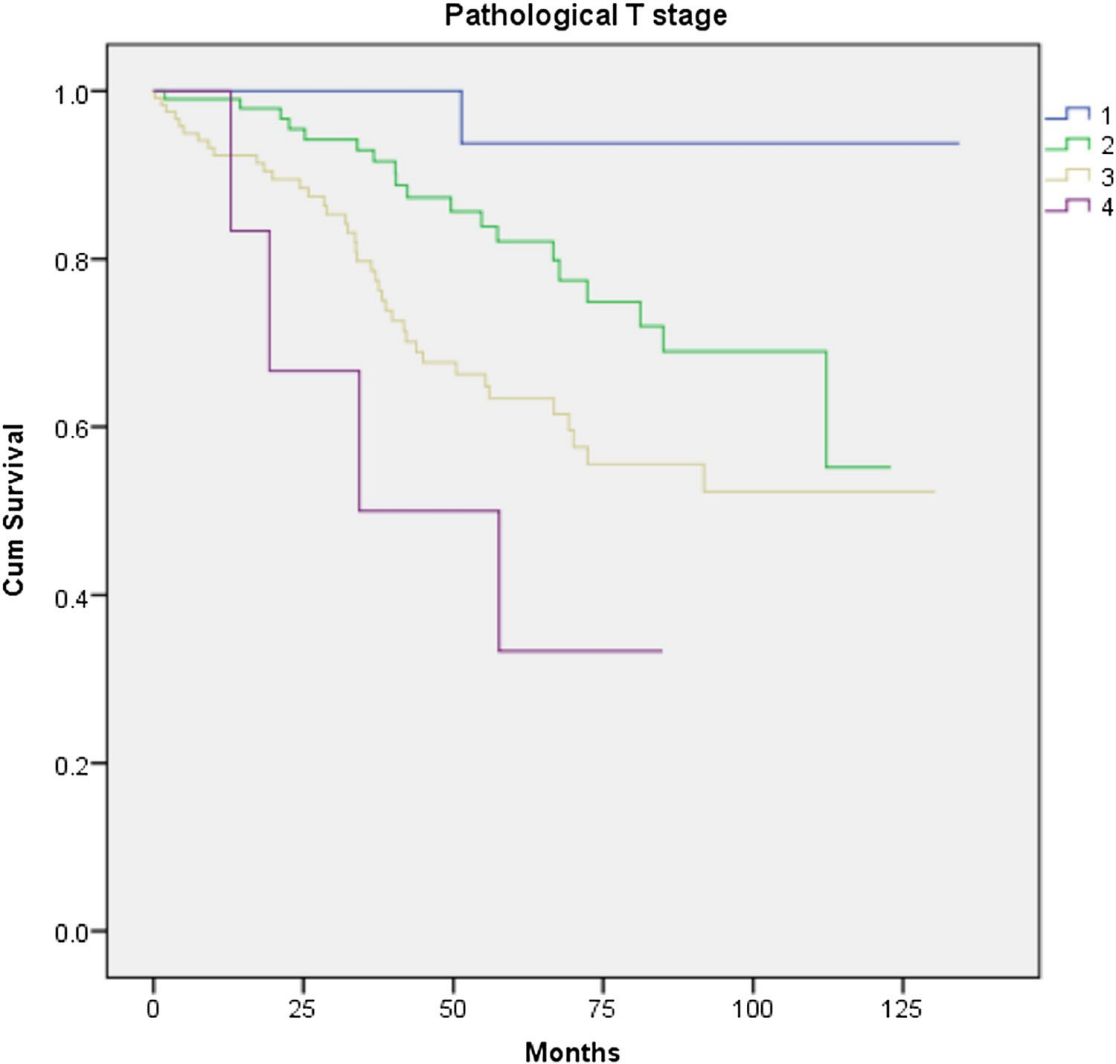
Cumulative survival for patients undergoing a curative resection for low rectal cancer according to T stage, log Rank = 0.001

**Fig. 7:**
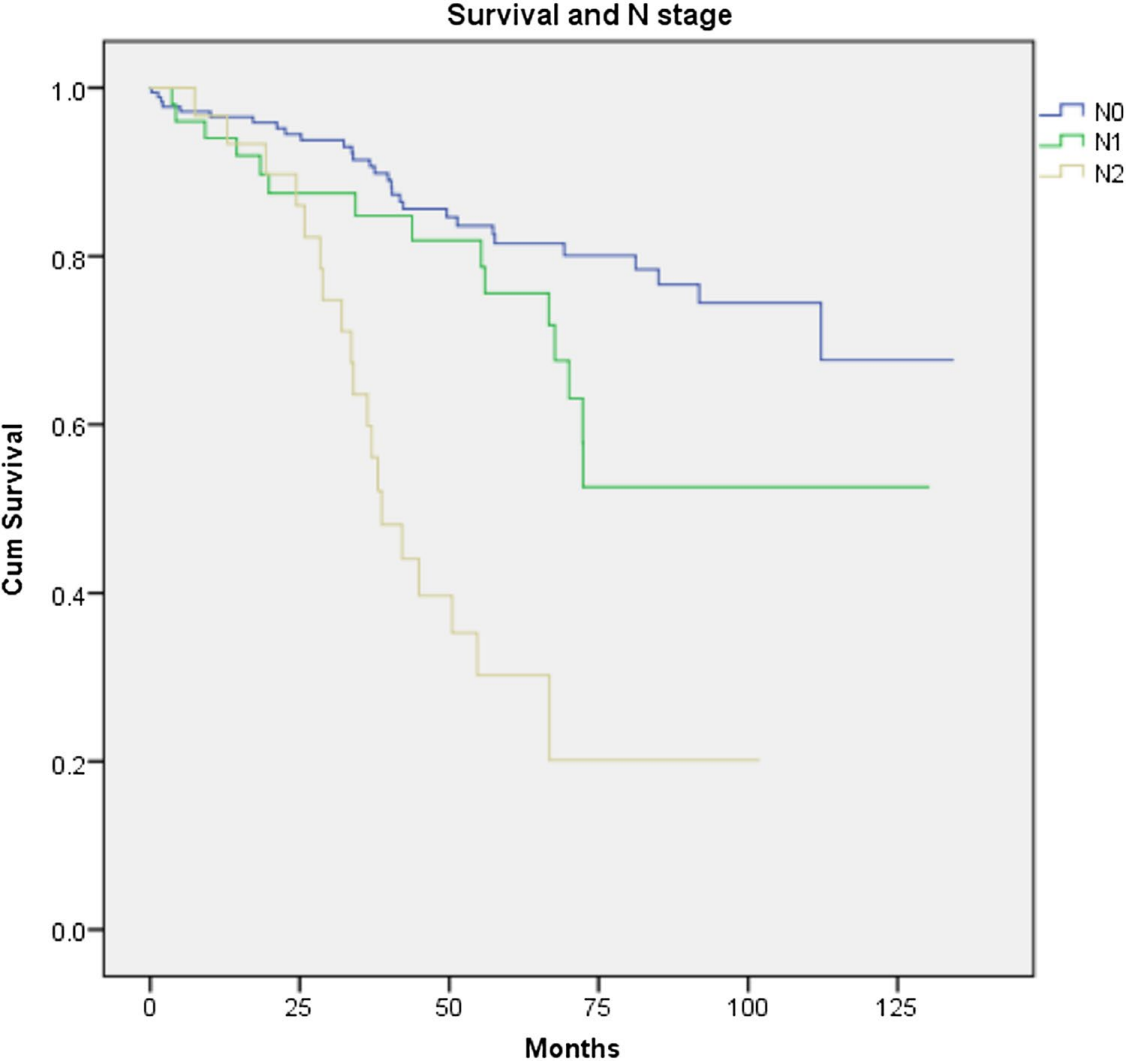
Cumulative survival for patients undergoing a curative resection for low rectal cancer comparing patients with N0, N1 and N2 stage, log Rank = 0.001

The overall recurrence rate (local and distant) was 17.6% in the LAR group compared to 23.9% for APER, p = 0.257. There was no difference either in the local recurrence or in the distant recurrence rate between the 2 groups (p = 1.000 and 0.215 respectively). There was also no significant difference in disease-free survival between the two groups (p = 0.455, Fig. 3). R1 resection was found to have a negative impact on survival and recurrence disease (Figs. 4 and 5). Furthermore, T stage and N stage was found to have a negative impact on patient survival (Figs. 6 and 7).

### Short term outcomes

Patients in the APER groups had a significantly longer length of primary hospital stay compared to LAR patients, p < 0.001. Overall, the post-operative complications rate was higher in the APER group compared to the LAR group, p = 0.028, see Table 3. Amongst both groups only one APER patient died of a myocardial infarction within 30 days of the operation. Ten LAR patients had to return to theatre, 6 for an anastomotic leak, 2 cases for abdominal sepsis and 2 cases for loop ileostomy related complications. In the APER group, again, 10 patients had to return to theatre, but for different reasons; 4 patients for stoma related complications, 3 cases for intra-abdominal sepsis and 3 patients needed a small bowel resection.

**Table 3:** Short-term post operative outcomes

Variables	LAR	APER	p value
Number (%)	170 (64.9)	92 (35.1)	
Overall complications	77 (45.3)	55 (59.8)	*0.028*
Clavien-Dindo classification			
I	21 (27.2)	4 (7.3)	
II	31 (40.3)	26 (47.3)	
IIIa	12 (15.6)	11 (20.0)	
IIIb	12 (15.6)	10 (18.2)	
IV	1 (1.3)	3 (5.4)	
V	0 (0.0)	1 (1.8)	
Urinary infection	16 (9.4)	5 (5.4)	0.343
Wound infection	17 (10.0)	25 (27.2)	*0.001*
Wound dehiscence	0 (0.0)	9 (9.8)	*< 0.001*
Intra-abdominal or pelvic collection	13 (7.6)	8 (8.7)	0.813
Percutaneous drainage	5 (2.9)	2 (2.2)	1.000
Anastomotic leak	22 (12.9)	NA	NA
High stoma out-put	13 (7.6)	NA	NA
Post-operative ileus	9 (5.3)	2 (2.2)	0.338
Post-operative bleeding	5 (2.9)	4 (4.3)	0.724
Re-operation	10 (5.9)	10 (10.9)	0.153
Readmission	25 (14.7)	16 (17.4)	0.595
Peri-operative mortality	0 (0.0)	1 (1.1)	0.351
Hospital stay, days, median (interquartile range)	7 (5–13)	12 (8–19)	*< 0.001*

Values in italics are statistically significant

Nominal variables expressed as absolute numbers with percentage in parenthesis

*APER* abdominoperineal resection, *LAR* low anterior resection

The LAR group had 22 patients who had anastomotic leakage (13%), of which 6 had to return to theatre, 2 had a percutaneous drainage and the remnant patients were treated conservatively. All 22 cases had been given a defunctioning loop ileostomy during the first operation.

**Fig. 8:**
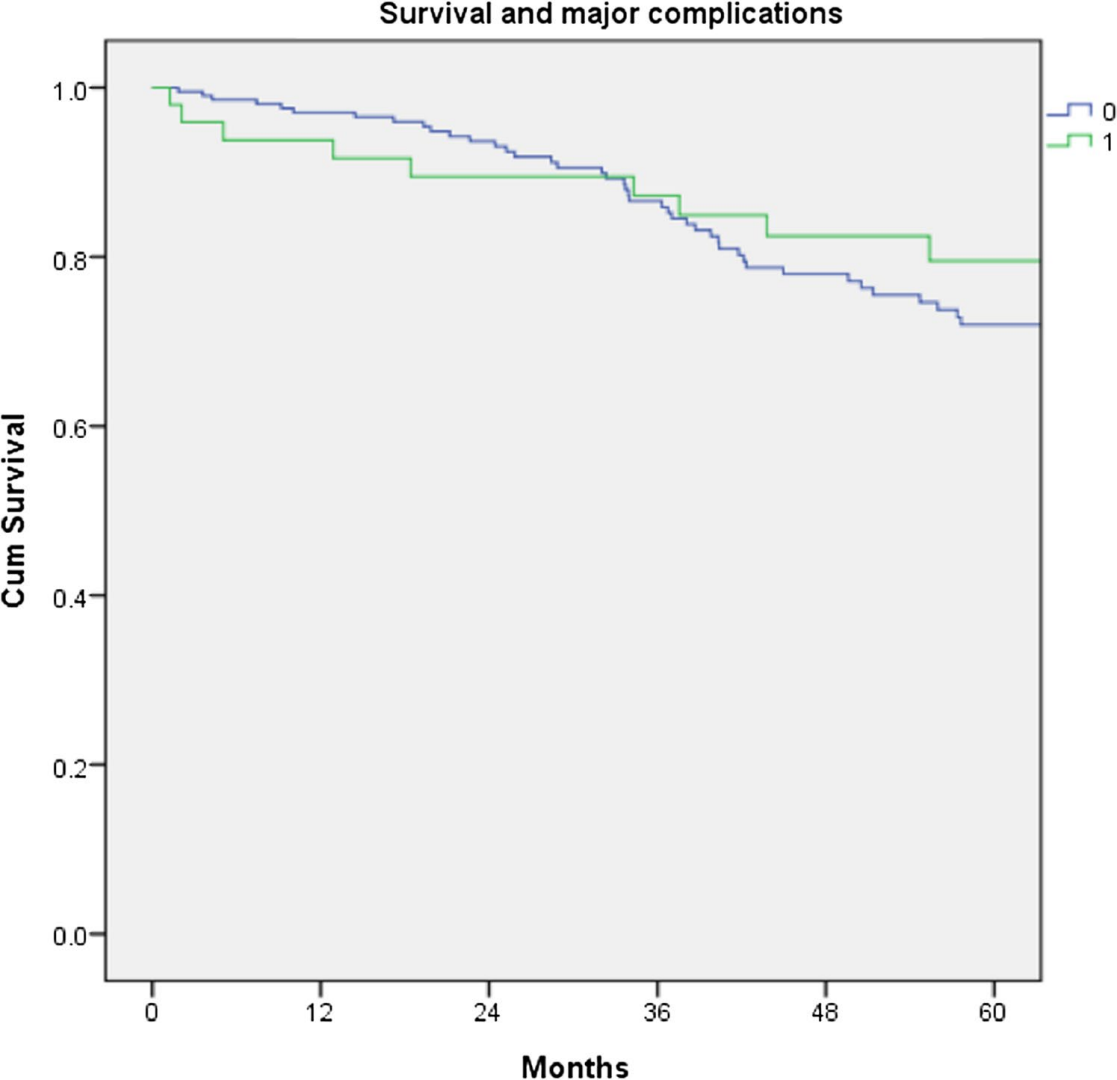
Cumulative survival for patients undergoing a curative resection for low rectal cancer comparing patients who had a major complication, log Rank = 0.442. 0 = no complication, 1 = major complication (Clavien Dindo Grade III/ IV)

Major complications (Clavien Dindo grade III and IV) occurred in 49 patients of the entire cohort (19%), but a major complication did not have a negative impact on long term survival, Fig. 8.

During last follow up, 99/170 (58.2%) in the LAR group had their loop ileostomy reversed. 19 cases (14%) from the APER group had developed a symptomatic parastomal hernia.

## Discussion

Rectal cancer patients still often experience surgical complications, regardless of the important progress made so far within both techniques and perioperative management. In this study, considerable short-term survival benefits in favor of the LAR group were achieved. Overall, post-operative complication rate was higher in APER group which was mainly caused by the high incidence of perineal wound failure and they had a significantly longer length of primary hospital stay compared to LAR patient (median 12 vs. 7 days, Table 3). The anastomotic leak rate in LAR was 13%.

The most common complication of APER is perineal wound failure and can be as high as 30% [16]. Anastomotic leakage is considered to be the major complication of restorative LAR with studies reporting an average leakage rate of 11–12% in high volume centers following rectal cancer surgery which is comparable with our AL rate [17]. However, it is demonstrated that the postoperative complications such as pelvic sepsis, urinary and sexual dysfunction, are higher in the APER group than in the LAR group [17].

In this study we achieved a sphincter-preservation rate of 50% in low rectal cancers and believe that this should remain a priority since only an average of 20% of patients in the APER group is satisfied with having a permanent colostomy [14]. In fact, patients undergoing APER have restrictions in their postoperative Quality of Life, such as body image, which can lead to altered social life [14, 15]. However, in elderly patients with more distal, more locally advanced disease that requires radiotherapy, as our data show, APER performed with appropriate skill, remains a safe option.

For the oncological outcome, the achievement of technical excellence in TME surgery at our center is reflected in the low local recurrence rates in both surgical groups when compared to other studies. For instance, a meta-analysis from 2015 suggested that compared to APER, LAR has better 5-year survival rates, lower CRM rates, less local recurrence and less complications [18].

CRM involvement is a recognized prognostic factor for local recurrence. Patients who undergo APER have shown to have a higher incidence of CRM involvement [18, 19] and has, unfortunately, not diminished with TME. The distance from the anal verge is related to the completeness of TME, because of the greater challenge of performing a perfect resection with adequate margins low down in the pelvis. TME surgery cannot always be carried out down to the levator muscles plane in APER because of the presence of a large tumor. CRM involvement in the APER specimens is often related to the removal of less tissue at the level of the tumor because of a different resection plane. However, the lower cancers elected for APER may be associated with a different pattern of lymphatic spread, which is not included in the tumor package harvested with TME [18, 19].

We believe that in selected low rectal cancer patients, APER is a better option than LAR. In current literature, despite rigorous methodology, the intrinsic limitation/bias of the studies should be considered, and conclusions interpreted with caution. The APER tumors are usually closer to the anal verge and more bulky. Although the patients in the LAR and APER groups are comparable in terms of age, tumor stage, and neoadjuvant treatment and the distribution of tumor stage, however, it would not be possible to eliminate this bias as bigger tumors would tend to undergo a Miles procedure, as sphincter saving would not be attempted. The extent of tumor spread in itself is therefore unlikely to account for the increased surgical margin involvement, consequent local recurrence and lower survival in the APR group.

## Conclusion

In conclusion, our single centre findings show that LAR has a similar oncological outcome for low rectal cancer when compared to APER. However, APER is associated with a higher rate of post-operative complications and longer hospital stay. A tailored approach suited to the individual patients needs supported by the multidisciplinary team should be recommended. 

## Data Availability

The datasets generated during and/or analysed during the current study are available from the corresponding author on reasonable request.
